# Assessment of 3 standards-based clinical decision support (CDS) tools in an academic electronic health record using Clinical Quality Language, CDS Hooks, and Fast Healthcare Interoperability Resources: a retrospective evaluation

**DOI:** 10.1093/jamiaopen/ooaf085

**Published:** 2025-07-30

**Authors:** Mark Isabelle, Ivan K Ip, Michael Bakhtin, Louise Schneider, Ali S Raja, Sayon Dutta, Adam Landman, Ronilda Lacson

**Affiliations:** Center for Evidence-Based Imaging, Department of Radiology, Brigham and Women’s Hospital, Boston, MA 02120, United States; Center for Evidence-Based Imaging, Department of Radiology, Brigham and Women’s Hospital, Boston, MA 02120, United States; Covenant Health, Inc, Tewksbury, MA, 01876, United States; Center for Evidence-Based Imaging, Department of Radiology, Brigham and Women’s Hospital, Boston, MA 02120, United States; Aluna Health, Cambridge, MA, 02142, United States; Center for Evidence-Based Imaging, Department of Radiology, Brigham and Women’s Hospital, Boston, MA 02120, United States; Department of Medicine, Harvard Medical School, Boston, MA, 02115, United States; Center for Evidence-Based Imaging, Department of Radiology, Brigham and Women’s Hospital, Boston, MA 02120, United States; Department of Emergency Medicine, Massachusetts General Hospital, Boston, MA, 02114, United States; Department of Emergency Medicine, Harvard Medical School, Boston, MA, 02115, United States; Department of Emergency Medicine, Massachusetts General Hospital, Boston, MA, 02114, United States; Department of Emergency Medicine, Harvard Medical School, Boston, MA, 02115, United States; Department of Emergency Medicine, Harvard Medical School, Boston, MA, 02115, United States; Department of Emergency Medicine, Brigham and Women’s Hospital, Boston, MA, 02115, United States; Center for Evidence-Based Imaging, Department of Radiology, Brigham and Women’s Hospital, Boston, MA 02120, United States; Department of Radiology, Harvard Medical School, Boston, MA, 02115, United States

**Keywords:** clinical decision support, information technology, health information exchange

## Abstract

**Objectives:**

To evaluate clinical decision support (CDS) of varying complexities and care settings represented using Health Information Technology (HIT) standards—Clinical Quality Language (CQL) for representing clinical logic and Fast Healthcare Interoperability Resources (FHIR) for health information exchange.

**Materials and Methods:**

This Institutional Review Board-approved, retrospective study was performed at an academic medical center (January 1, 2023-December 31, 2023). Recommendations extracted from patient-centered outcomes guidelines were translated into standardized syntax (SNOMED CT) and representations (CQL, FHIR). Clinical decision support Hooks applications were developed for: CDS1—provides education for emergency department (ED) patients with venous thromboembolism; CDS2—recommends CT pulmonary angiogram in ED patients with suspected pulmonary embolism (PE) and uses FHIR Questionnaire resources for representing interactive content; CDS3—recommends mammography/breast magnetic resonance imaging surveillance in outpatients with breast cancer history. We randomly selected 50 ED patients with suspected PE and 50 outpatients undergoing breast imaging surveillance. We compared outcomes of false-positive alerts and the accuracy of CDS1, the more complex CDS2, and CDS3 for outpatients.

**Results:**

Clinical decision support Hooks applications used CQL logic for trigger expressions and logic files and provided recommendations to ED and outpatient providers. CDS1 had a false-positive alert and accuracy of 11.1% and 98%, respectively, not significantly different from CDS2 (0.0% false-positive alerts, *P* = .33 and 96% accuracy, *P* = .56) or from CDS3 (0.0% false-positive alerts, *P* = .15 and 100% accuracy, *P* = .31).

**Discussion:**

Health Information Technology standards can represent recommendations of varying complexities in various care settings.

**Conclusion:**

The potential to represent CDS using standardized syntax and formats can help facilitate the dissemination of CDS-consumable artifacts.

## Background and significance

Well-implemented clinical decision support (CDS) can improve health-care processes,^1^ including medical imaging.^2–5^ However, if poorly executed, CDS can create interruptive electronic health record (EHR) workflows and low-value clinical alerts, potentially contributing to physician burnout,^6^^,^^7^ with little or no impact on imaging utilization or appropriateness. Integrating patient-specific parameters and consideration of human factor design principles are important in developing CDS that provide value to users.^8^^,^^9^ Additionally, the strength of the evidence underlying CDS recommendations may enhance or impede the usability of CDS alerts^10^^,^^11^ and represents an important attribute in assessing practice guideline trustworthiness.^12^ High rates of false-positive alerts (ie, low specificity) also contribute to low-value CDS.

The Harvard Medical School Library of Evidence (HMS-LOE) has developed a publicly available online “evidence library”^10^ to provide a repository of medical evidence that can be utilized in CDS systems, including for the imaging domain.^13^ However, graded evidence must be represented in a more structured format for use as CDS. In particular, standard formats can help facilitate interoperability and public dissemination of CDS. Therefore, we aimed to evaluate accuracy and false-positive rates (FPRs) for CDS of various complexities and combinations for different care settings, represented using Clinical Quality Language (CQL)^14^ and Fast Healthcare Interoperability Resources (FHIR).^15^

## Materials and methods

### Study setting and human subjects approval

We conducted a 12-month, retrospective cohort study (January 1, 2023-December 31, 2023) at a tertiary academic medical center with an emergency department (ED) and an outpatient network spanning 183 practices and 1200 physicians. The study institution uses Epic (Epic Systems Corporation) as its EHR across all care settings, and imaging orders are placed via order entry within Epic. The Institutional Review Board approved the study and waived the requirement for informed consent.

### CDS applications

We aimed to develop 3 CDS Hooks applications based on preexisting patient-centered outcomes guidelines.^16–19^ We specifically focused on CDS to address the need for radiologic imaging as the role of radiologic imaging in screening for and diagnosing disease has greatly expanded in the last 2 decades, with an estimated 400 million imaging tests conducted annually.^20^^,^^21^ Each unit of evidence from the guidelines was previously extracted and represented in the HMS-LOE as a Clinical Evidence Logic Statement (CELS) of “If-Then” form in a semistructured layer (level 2) based on a 4-layered framework (ie, levels 1-4) for knowledge representation.^22^ In addition, the CELS were graded using a system developed by the Oxford Centre for Evidence-Based Medicine^23^ and the United States Preventive Service Task Force system.^24^ The system has 5 levels: level 1 includes validating cohort studies with good reference standards and studies with findings whose specificity or sensitivity is so high to rule in/out a diagnosis. Level 2 includes exploratory cohort studies with good reference standards. Level 3 includes studies that are either nonconsecutive or have no consistently applied reference standards. Level 4 includes case-control studies and those with poor or nonindependent reference standards. Level 5 refers to expert opinions. The evidence sources were purposefully selected to generate CDS of varying complexities and for various care settings.

CDS1 was a simple CDS Hooks application utilizing CQL logic for the trigger expression and logic files. It aimed to provide educational materials to ED patients with suspected pulmonary embolism (PE) and those with previous history of venous thromboembolism (VTE). CDS1 emphasized patients’ preference for learning about thromboembolism symptoms, risk factors, prevention, and complications in the context of a doctor-patient encounter.^16^ Educating patients regarding PE can potentially minimize those requiring further management, including testing and hospitalizations.

CDS2 was a more complex CDS Hooks application with the same trigger for recommending use of computed tomography pulmonary angiogram (CTPA) in ED patients with suspected PE. CDS2, however, proceeds to evaluate other elements in the EHR (eg, D-dimer) before returning a CDS Hooks Card, one of which additionally used FHIR Questionnaire resources to represent interactive content for providers to assess patient risk for PE. Given that the use of CTPA to diagnose PE is increasing,^25^ and that evidence-based guidelines for managing patients suspected of having PE exist, it is an excellent target for evaluating the impact of CDS, as integrating CDS for CTPA has the potential to decrease potentially unnecessary diagnostic imaging examinations while minimizing patient risk.^26^^,^^27^

CDS3 was based on a recommendation in a PCORI-funded grant^19^ for using breast magnetic resonance imaging (MRI) (instead of or in addition to mammography) in outpatient clinics for diagnosing a second breast cancer in women previously diagnosed with breast cancer and addresses women at highest risk for breast cancer. It emphasizes the use of surveillance breast MRI which resulted in increased biopsy and subsequent cancer detection rate relative to the use of mammography alone. Given that only a minority of these women receive breast MRI, the potential impact of this guideline is huge.

### CDS design principles and process

We used human factor principles to inform the design of the CDS applications, including minimizing false alarms, and the format of textual information and alert display.^8^ We also included other sociotechnical factors from the Systems Engineering Initiative for Patient Safety model. Systems Engineering Initiative for Patient Safety is based on a macroergonomics works system model that integrates Donabedian’s Structure-Process-Outcome framework to improve quality and provides a comprehensive conceptual framework for applying systems engineering.^9^^,^^28^^,^^29^ The model consists of 3 major components: the “Work System,” the “Process of Care,” and “Team Outcomes.” The “Work System” component encompasses 5 interconnected elements: person (eg, providers); tools and technology (eg, CDS); the external environment (eg, the physical environment); tasks (eg, task complexity), and organization (eg, organizational support). We specifically focused on persons, tools, and tasks.

Data from 26 patients was intentionally selected to design CDS1 and CDS2, individually and in combination. Data from an additional 14 patients were selected for CDS3. The selection included a range of patient scenarios when the CDS should, and should not, trigger. For instance, patients chosen for CDS2 included those at varying risks for PE, those with and without D-dimer testing results present, and those with and without exclusion criteria from the CDS, including patients under 18 years, pregnant patients, and those with estimated glomerular filtration rate (eGFR) < 30 or an allergy to iodinated contrast (contraindications for CTPA).

### Implementing CDS in the EHR

After design, we implemented each CDS Hooks application in the Epic Innovation environment (Epic INV), a separate nonproduction Epic environment deployed at the study institution. We first created a web application-type integration record or Field Device Integration for Substitutable Medical Applications and Reusable Technologies (SMART) on FHIR, a standard for application development for use with EHRs. We used the default SMART on FHIR authentication method for a new application ID. The application was launched in Epic Workspace from September 2024 to November 2024.

All patients were recreated in the nonproduction (ie, Epic INV) test environment in the same way it is done in Production (ie, using a “Create Patient” option). We entered matching demographic information (eg, age, race, sex) with patients queried from Production. All other patient history gets added into corresponding sections in Epic (eg, surgeries into surgical history section, contrast allergies under the Allergy section). Laboratory results (eg, D-dimer, eGFR) were entered as External Results using a “Enter External Results” option in Epic.

A flowchart of each CDS application and its expected responses were created (Figure S1A-C). We noted whether the CDS would fire appropriately with an appropriate CDS Hooks Card, thereby minimizing false-positive alerts, using the 40 patients used for the CDS design. For instance, if a patient has current VTE, the CDS displays the link for VTE educational materials. However, when an elevated D-dimer is noted in the EHR, the CDS terminates and allows a provider to proceed without further action. In addition, we assessed feedback from 4 experts—2 ED physicians and 2 primary care physicians, who were not involved in the original design of the CDS. This feedback was collected using a questionnaire with comments that can be provided as free text (Figure S2) and was considered in iterative cycles of refinement of the CDS applications before implementation. Most common critiques were grouped into themes, according to CDS design principles (Table S1). These are described further below in Sociotechnical Factors in CDS Design.

### Study population and data collection for CDS evaluation

We retrospectively evaluated each of the 3 CDS applications. The eligible study population included all adult patients from Epic’s Enterprise Data Warehouse (EDW) who had: (1) an order for CTPA for suspected PE at the ED or (2) an order for a surveillance breast imaging examination (breast MRI or mammography) in outpatient women with a previous history of breast cancer or ductal carcinoma in situ (DCIS), during the study period. The EDW was queried to identify all possible patients and then we randomly selected 100 patients from this set; 50 from each of the 2 groups of patients. This sample size was calculated to detect whether there was a greater than 27% difference in the FPR of CDS from our previously defined baseline of 15% based on an increase in the proportion of patients triggering a CDS alert between 16.5% and 37.0% in previous studies,^30^^,^^31^ with a 95% CI level and 80% power. These patients were subsequently used in the FHIR calls in Epic INV.

The Institutional Research Data Warehouse, populated by Epic, was used to extract patient-specific features including age, sex, race, and ethnicity. For CDS1 and CDS2, we also extracted risk factors for PE, including previous history of PE or VTE. Other information extracted from the data warehouse included D-dimer results, if present, pregnancy status, eGFR, and iodinated contrast allergy. For CDS3, we extracted previous breast cancer and cancer stages. Manual review was conducted to verify whether the CDS fired appropriately based on EHR review and the variables extracted. Clinical decision support inclusion and exclusion criteria, as well as logic to activate a CDS Hooks Card are shown in Table S2.

### Outcome measures and statistical analysis

As coprimary outcomes of CDS performance, we measured the false-positive rate (FPR) and accuracy for each CDS alert. False-positive rate is defined as the number of false-positive alerts out of all patients who should not have received an alert (ie, 1—specificity). Accuracy is defined as the number of true positives and true negatives out of all patients analyzed. We compared the false-positive alert rate and accuracy for the CDS1 application compared to the more complex CDS2 application. We also compared the 2 outcomes for CDS1 with CDS3 to assess scalability in CDS designed for another care setting. Chi-square and Fisher’s Exact tests were used to compare accuracy and FPRs. Finally, we also reported the precision and recall (ie, true positive rate) for all 3 CDS applications.

## Results

### CDS development

The CDS applications were developed using CDS Hooks. CDS Hooks is an HL7 Standard that is a RESTful, JavaScript Object Notation-based web service specification that uses FHIR to exchange patient data. We used CQL logic for the trigger expression and logic files (Figure S3A-C). We used implicit value sets in the CQL expressions (https://build.fhir.org/valueset.html#implicit) that allow us to use the Snomed or International Classification of Diseases, Tenth Revision concept hierarchies to include a large group of conditions. An Epic “order-select” trigger triggers the workflow for CDS Hooks. Once triggered, the web service then requests the clinical data it needs from the EHR. The CDS application is using a “prefetch template” that instructs Epic to attach selected FHIR data elements along the initial call. This eliminates the need for the service to call back into the FHIR server before obtaining clinical data, enhancing performance.

The CDS Hooks applications evaluate the content using an internal registry of CQL rules and provide the appropriate Card reply, when applicable. In the case of CDS1, the Card provides a link to a separate URL. In the case of CDS3, it gives recommendations in text. Finally, the Card provides a link to a FHIR Questionnaire resource. The FHIR Questionnaire retrieves patient data and uses it to answer questions, when available. Otherwise, it asks for additional PE risk factors so providers can assess PE risk and receive a tailored recommendation for CDS2.

**Figure 1.**
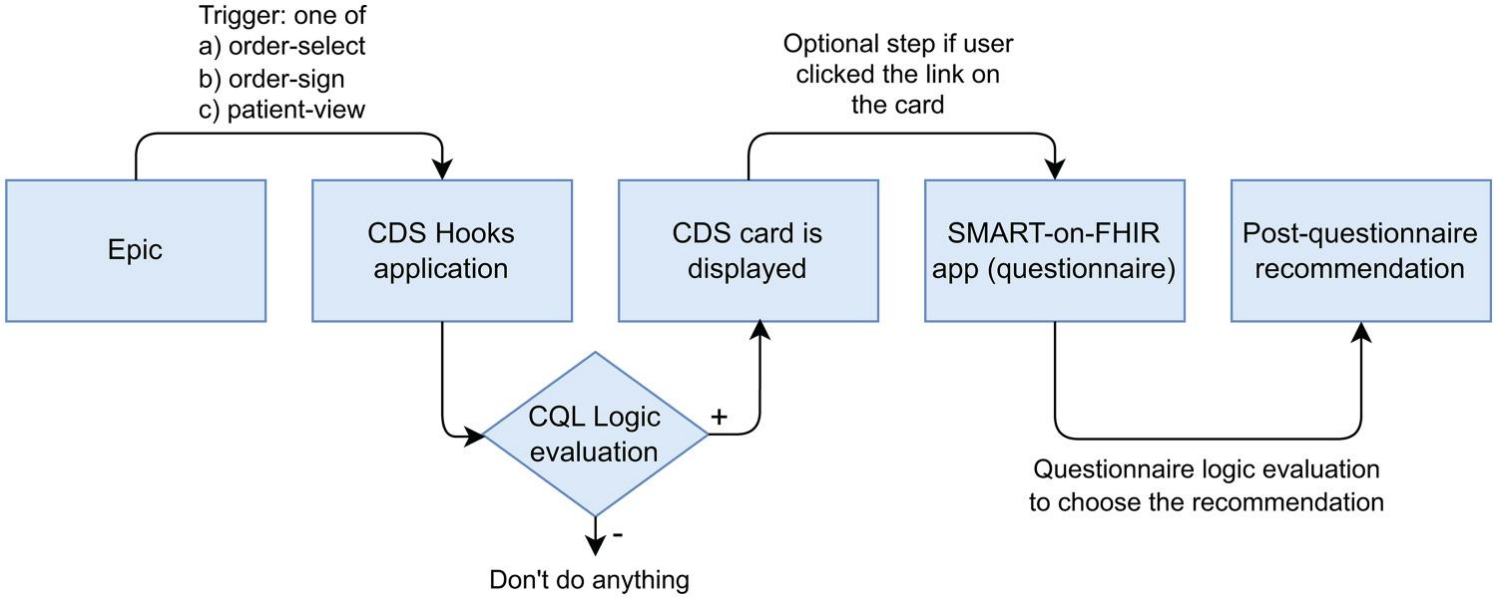
CDS application workflow. Abbreviation: CDS, clinical decision support.

This workflow is illustrated in Figure 1.

### CDS1: recommendations for providing educational materials on VTE prevention

Clinical decision support artifacts (using CQL and FHIR) have been published in CDS Connect^32^ based on a published study.^16^ The recommendation aims to provide educational materials to patients when seen by a physician at the ED for suspected PE. When a physician orders a CTPA while the CDS is in place and when appropriate, the CDS Card provides a link to educational materials in another URL (Figure S1A),^33^ which the physician can discuss with the patient or for another provider to print out for the patient. This is graded as level 4 evidence by the HMS-LOE.

### CDS2: recommendations for diagnostic evaluation of suspected PE with CT pulmonary angiogram

Clinical decision support artifacts are published in CDS Connect^34^ based on Wells’ criteria for PE management (Figure S1B).^17^^,^^18^ The CDS aims to optimize the appropriate use of CTPA for evaluating suspected acute PE. Specifically, it is triggered by an order for CTPA to evaluate patients with suspected PE. This CDS should only be used in adult patients (18 years or older). In addition, this CDS has not been validated for use in pregnant patients. Caution should be applied in patients with severe allergy to iodinated contrast and patients with eGFR < 30 for whom alternative imaging should be considered. This is graded as level 2 evidence by the HMS-LOE.

### CDS3: recommendation for surveillance breast imaging in women with personal history of breast cancer

Clinical decision support artifacts are published in CDS Connect^35^ to ensure patient preferences are included in the decision process for choosing mammograms or breast MRIs in outpatients with a history of breast cancer, specifically in stages 1-3 and DCIS.^19^ It is intended for women with a personal history of breast cancer for whom either a mammogram or breast MRI is ordered for surveillance, which act as the triggers. Textual information is provided to inform providers to assess patient preferences given the limitations of either examination (Figure S1C). Women with stage 4 breast cancer are excluded from this recommendation. This is graded as level 3 evidence by the HMS-LOE.

### Data availability

The graded pieces of evidence underlying this article are available in the Harvard Medical School Library of Evidence (https://libraryofevidence.med.harvard.edu/) upon request. Other CDS artifacts are available in CDS Connect.^32^^,^^34^^,^^35^

### Sociotechnical factors in CDS design

We utilized results from the questionnaire administered to 4 experts in assessing the CDS. Results of the questionnaire for physician’s attitude toward the CDS are shown in Figure S2, indicating a positive response to the CDS (means 6-7; scale 1-7) for positive impact on workload, ease of use, reducing diagnostic errors, adequate training, and improving quality of care. Similarly, responses to questions regarding physician satisfaction with the CDS indicate that physicians were very satisfied with the CDS (means 6-7; scale 1-7). Textual comments (Table S1) were incorporated into the iterative design below.

#### Person factors: minimizing false-positive alerts for providers and incorporating patient-specific factors

False alarms increase workload and may cause distraction and alert fatigue.^36^^,^^37^ This would then contribute to decreased performance.^38^ We, therefore, tested the CDS with data from various patients to ensure that false-positive alerts are minimized for providers. We accomplished this by ensuring patient-specific data are accessed by the web service and exclusions are appropriately determined so they do not cause false alerts. We were unable to operationalize 2 exclusions—pregnant patients where pregnancy was not noted in the problem list (for CDS2) and stage 4 breast cancer (for CDS3). Cancer staging was not accurately documented in the problem list, thus making it difficult to exclude. We therefore chose to include all cancers, which may lead to false alerts for patients with previous stage 4 breast cancer (who should be excluded from CDS3).

For the most part, we captured all other patient-specific exclusions (eg, age < 18 or minors), which decreased the number of false-positive alerts in the test data. Minimizing false-positive alerts would reduce the overall number of alerts and the frequency with which they activate, a key principle for alerts and alarms,^8^ and a goal for providers.

#### Tool factors: optimizing visual design and textual information

**Figure 2.**
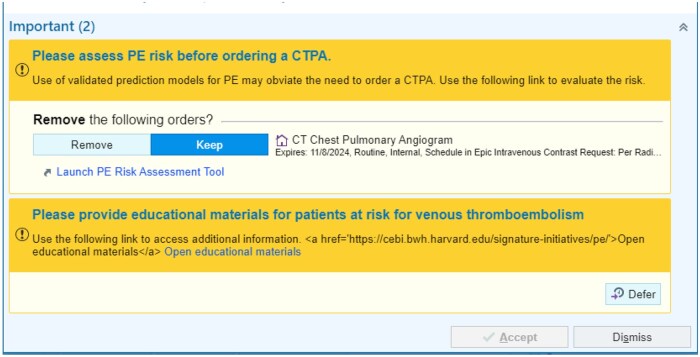
Example of 2 CDS firing. Abbreviation: CDS, clinical decision support.

Visual alerts were prioritized, and color was used to make the alert content distinctive on the Epic best practice alert.^8^ The CDS titles and goals were in a bright yellow header, distinct from the white background of the typical EHR notes. Clinical decision support titles and goals were at the top of the alerts, along with textual information and links to URLs for additional information. These were on a yellow background, to separate them from the main body of the alert (in lighter yellow) where providers could cancel or keep an ordered examination (Figure 2).

More importantly, we focused on the textual content of the information presented. It has been shown that definitive rather than passive or probabilistic statements can increase the effectiveness of alerts. Thus, we used definitive verbs, such as “Please assess PE risk…” or “Please provide educational materials….”^39^ This is preferred over a more passive statement, such as “Risk assessment can be performed…” or a probabilistic statement such as “Risk may be assessed in some patients.” Second, the order of words reflects the expected order of actions recommended. For instance, “Please assess PE risk before ordering a CTPA.”^8^ Finally, we focused on the brevity and clarity of the recommendations presented.

Finally, we assessed the design for firing combination alerts (with more than 1 CDS firing), as in Figure 2. When CDS1 and CDS2 fired simultaneously, we prioritized CDS2 by placing it at the top of CDS1. We wanted providers to focus more on this alert than the one below, which although educational does not impact diagnostic examination ordering.

#### Task factors: simplifying tasks and reducing number of screens

**Figure 3.**
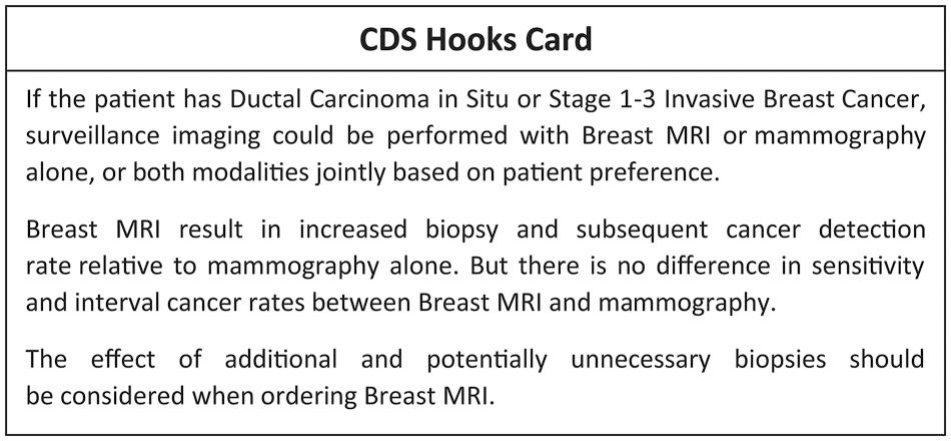
Displayed information for providers.

We aimed to minimize providers’ efforts to review CDS alerts. Alerts that require acknowledgement before the user can proceed were kept to a minimum. Specifically, for CDS3, a FHIR Questionnaire for patients with a prior history of breast cancer was originally designed on a separate URL. However, it was deemed more efficient to display the textual data in the first display (Figure 3), thus minimizing the task complexity for providers and facilitating the process of proceeding from the CDS alert.

### Study cohort

Table 1 includes the demographic information of the 2 sets of 50 patients each, randomly selected for CDS application evaluation.

**Table 1.** Demographics of patients for CDS application evaluation.

Characteristics of patients with an order for CTPA	Number of patients, *n*=50 (%)
**Age**	Mean = 62 (range 30, 97)
**Sex**	
Male	23 (46%)
Female	27 (54%)
**Race**	
White	36 (72%)
Black	5 (10%)
Other	6 (12%)
Unavailable	3 (6%)
**Ethnicity**	
Hispanic	10 (20%)
Non-Hispanic	40 (80%)
**Contrast allergy**	3 (6%)
**eGFR < 30**	4 (8%)
**Prior DVT/PE**	8 (16%)
**Characteristics of patients with an order for breast imaging**	**Number of patients, *n*=50 (%)**
**Age**	Mean = 61 (range 37, 82)
**Race**	
White	36 (72%)
Black	3 (6%)
Other	7 (14%)
Unavailable	4 (8%)
**Ethnicity**	
Hispanic	1 (2%)
Non-Hispanic	49 (98%)
**Breast imaging modality ordered**	
Mammography	49 (98%)
Breast MRI	1 (2%)
**Breast cancer stage**	
DCIS	12 (24%)
Stage 1	20 (40%)
Stage 2	13 (26%)
Stage 3	4 (8%)
Stage 4	0 (0%)
Unavailable	1 (2%)

Abbreviations: CDS, clinical decision support; CTPA, computed tomography pulmonary angiogram; DCIS, ductal carcinoma in situ; eGFR, estimated glomerular filtration rate; DVT, deep vein thrombosis; PE, pulmonary embolism; MRI, magnetic resonance imaging.

The mean age for patients seen in the ED with an order for CTPA for suspected PE is 62 years, similar to the mean age of 61 years for patients seen in the ambulatory clinic for breast imaging, and 72% of patients in both groups were White. However, 20% of patients in the ED were Hispanic, whereas only 2% were Hispanic in the ambulatory cohort of patients for breast imaging in women with previous history of breast cancer.

### False-positive rate and accuracy

Table 2 includes the FPR, accuracy, precision, and recall for each CDS application. For patients with suspected PE, the CDS for providing educational materials (CDS1) had a false-positive alert rate and accuracy of 2% and 98%, respectively. This was not significantly different from the CTPA CDS (CDS2), with 0.0% false-positive alert (*P* = 1.00) and 96% accuracy (*P* = 1.00). It is also not significantly different from the breast imaging CDS (CDS3), which had an accuracy of 100% (*P* = 1.00).

**Table 2.** False positive rate (FPR), accuracy, precision, and recall for each CDS application.

CDS	True positives	False positives	False negatives	True negatives	FPR	Accuracy	Precision	Recall
**CDS1**	8	1	0	41	1/42 (2%)	49/50 (98%)	8/9 (89%)	8/8 (100%)
**CDS2**	18	0	2	30	0/30 (0%)	48/50 (96%)	18/18 (100%)	18/20 (90%)
**CDS3**	50	0	0	0	0/0 (n/a)	50/50 (100%)	50/50 (100%)	50/50 (100%)

Abbreviation: CDS, clinical decision support.

Precision=true positive/(true positive+false positive).

Recall=true positive/(true positive+false negative).

## Discussion

Health Information Technology standards can be used to represent 3 recommendations for CDS with various complexities—providing educational materials for patients with suspected PE with previous VTE, recommending the use of CTPA in patients with suspected PE, and using mammography and breast MRI for breast cancer surveillance in outpatients with prior history of breast cancer. Substitutable Medical Applications and Reusable Technologies on FHIR apps have been used with CDS Hooks to increase CDS utilization in a previous study.^40^ The second CDS, in addition to using CDS Hooks for invoking CDS from the clinical workflow and CQL for representing clinical logic, also uses FHIR Questionnaire resources for representing interactive content. This provides an additional layer of complexity to the representation, albeit still using HIT standards. The accuracy and false-positive alert rate between the CDS did not vary significantly despite the added complexity.

We also assessed the scalability of standards for evaluating CDS designed for an outpatient care setting compared to the ED. The accuracy and FPR of the CDS that was used for breast cancer surveillance in patients with prior history of breast cancer also did not vary significantly from the CDS used for ED patients. The CDS can trigger accurately after preliminary testing with patients and experts to ensure that CDS would fire appropriately, thereby minimizing false-positive alerts that have led to alert fatigue, leading physicians to bypass future alerts.^41^^,^^42^ Incorporating sociotechnical factors in CDS design, specifically person factors,^8^ minimized false-positive alerts.

In addition to Person factors, Tool and Task factors are important for improving provider experience.^8^ In our case, the use of definitive rather than probabilistic statements in the textual content provided was an important factor that has been shown to strengthen CDS effectiveness. Furthermore, in addition to textual information, the prioritization of the alerts is an effective strategy for improving CDS acceptance, as previously reported.^43^^,^^44^ In our case, we prioritized CDS2 by placing it at the top of CDS1 when both fired simultaneously. This prioritization was designed to enable providers to focus more on an alert that will impact diagnostic examination ordering. Our experts agreed with optimizing Tool factors by optimizing alert prioritization and textual content.

This study was limited by the number of CDSs assessed and its retrospective design. Future studies will focus on prospective assessment of these CDS tools in a clinical setting with a larger population for testing to assess the accuracy, FPRs, user acceptance, and response to recommendations. Second, artificial intelligence (AI) will likely change how CDS is utilized in health care in many ways. If AI actively triggers CDS, provider interaction and the use of HIT standards for such use cases must be explored. Third, we did not assess CDS scalability in institutions other than where the CDS was designed and developed, potentially limiting generalizability. The CDS3 cohort was comprised of 98% non-Hispanic women, also impacting generalizability. These will be assessed in future dissemination studies.

In conclusion, it is possible to use HIT standards to represent recommendations with various complexities and for different care settings embedded within an EHR. Using sociotechnical factors in CDS design is vital in improving provider experience. A multifaceted approach, with CDS based on high-quality graded evidence, implemented using AI to enhance workflow efficiency and HIT standards will potentially improve CDS dissemination and acceptance. This will enhance CDS utilization, thus improving conformance to clinical guidelines and recommendations.

## Supplementary Material

ooaf085_Supplementary_Data
