# Supplementary material for: Assessment of 3 standards-based clinical decision support (CDS) tools in an academic electronic health record using Clinical Quality Language, CDS Hooks, and Fast Healthcare Interoperability Resources: a retrospective evaluation
Source: JAMIA Open. 2025 Jul 30;8(4):ooaf085. doi: 10.1093/jamiaopen/ooaf085 (PMC12309839; doi:10.1093/jamiaopen/ooaf085)
Supplement: ooaf085_Supplementary_Data [file ooaf085_supplementary_data.docx]

**Supplemental Figures 1A-C: Clinical Decision Support (CDS) Flowcharts for each CDS Hooks Application and Expected Responses**

1A: CDS 1 - Recommendations for Providing Educational Materials on Pulmonary Embolism and Venous Thromboembolism (VTE) Prevention


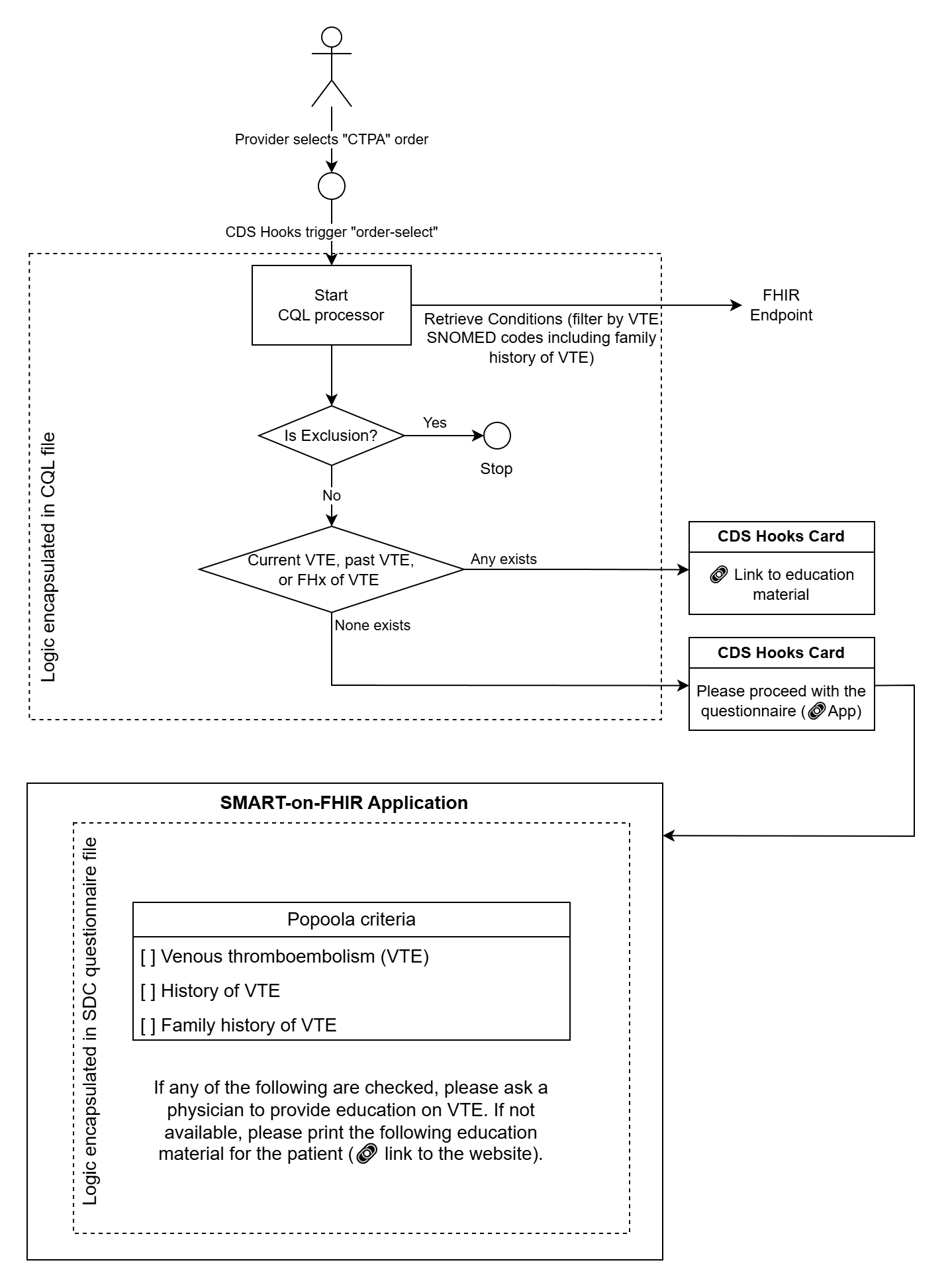


CTPA=CT pulmonary angiogram; FHx=family history

1B: CDS2 - Recommendations for Diagnostic Evaluation of Suspected Pulmonary Embolism (PE)


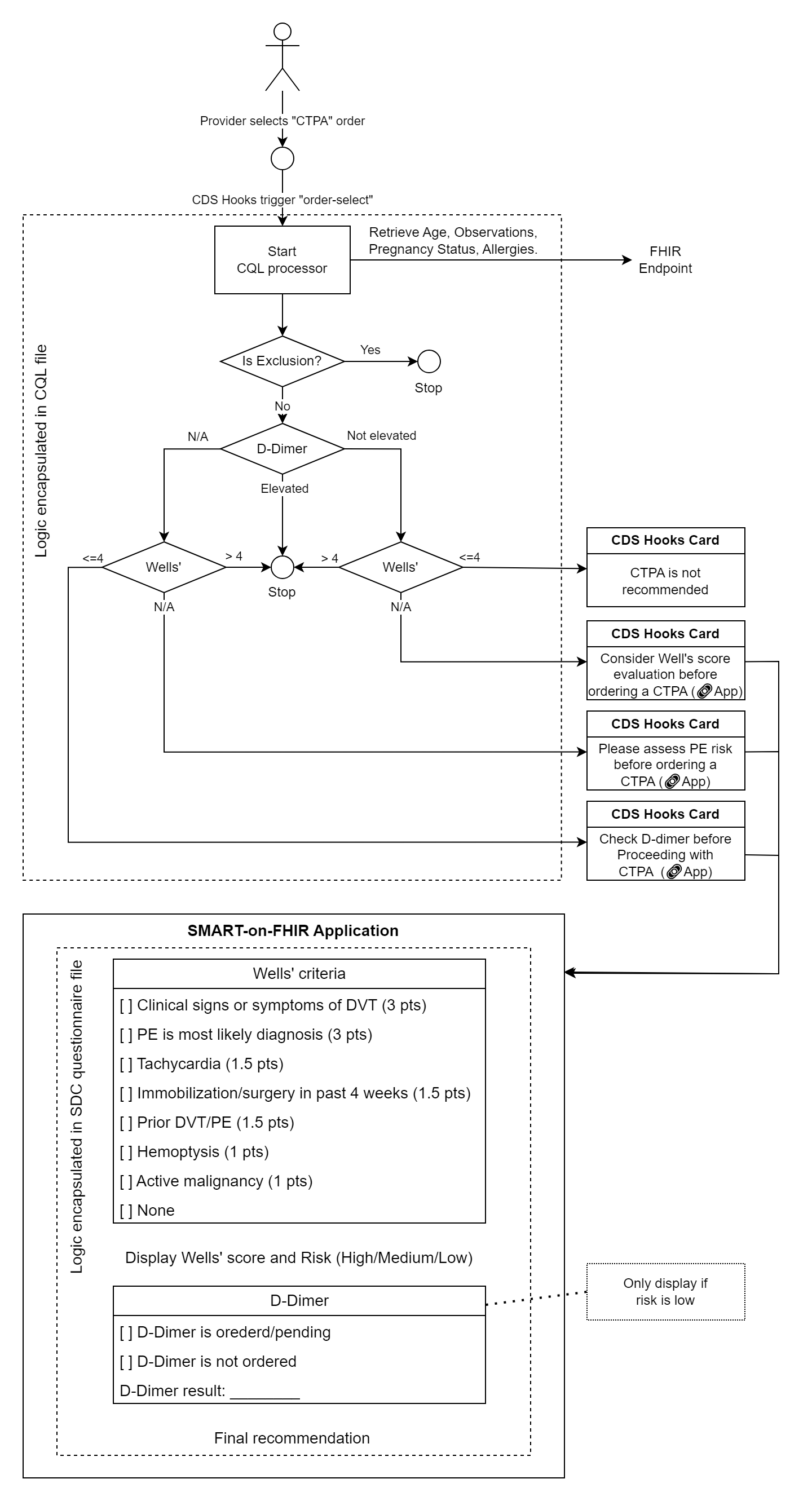


CTPA=CT pulmonary angiogram; DVT=deep venous thrombosis

1C: CDS3 - Recommendation for Surveillance Breast Imaging in Women with Personal History of Breast Cancer (BrCa) or Ductal Carcinoma in Situ (DCIS)


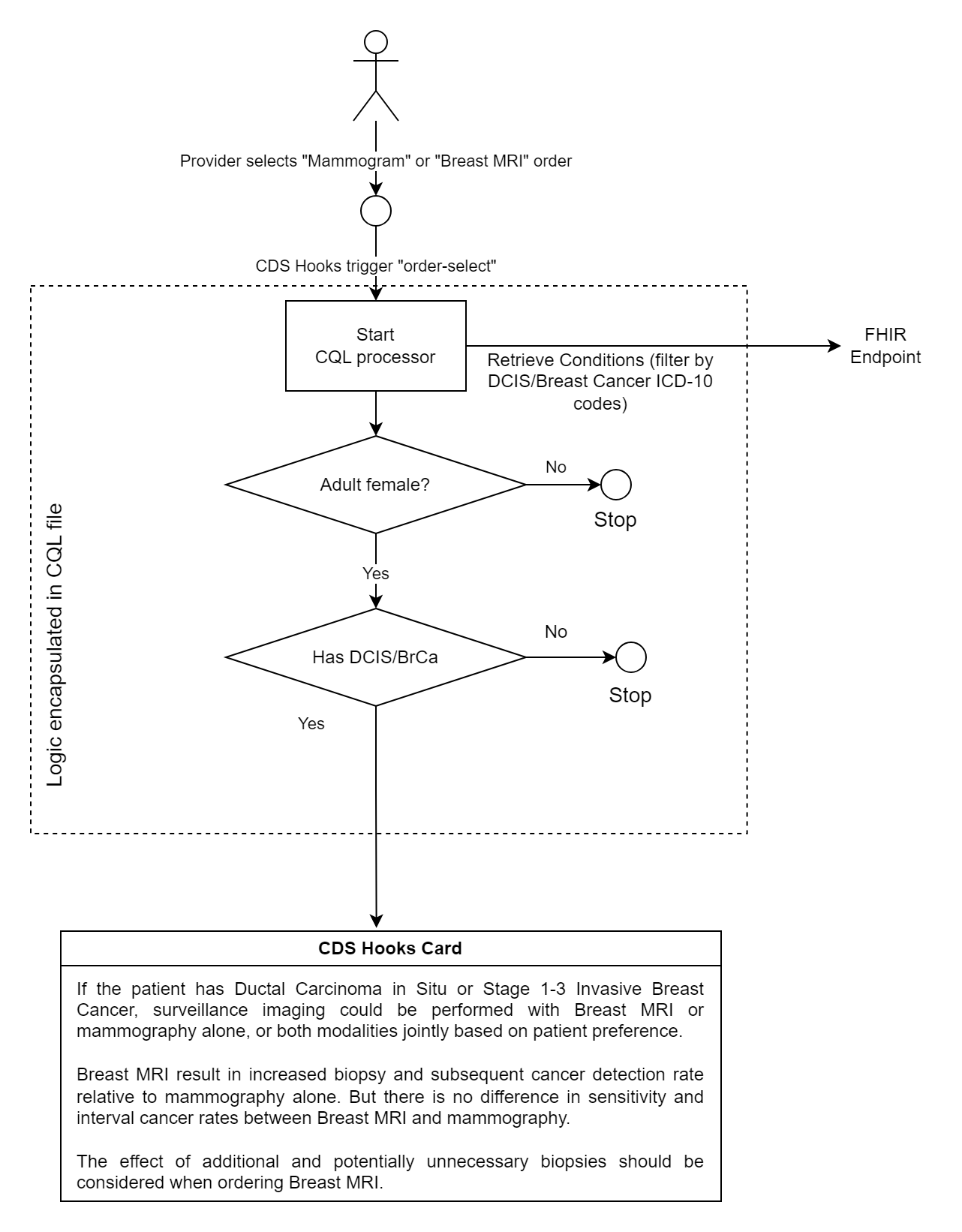


**Supplemental Figure 2: Questionnaire used for Clinical Decision Support (CDS) Assessment and Mean Responses (in bold)**

| **Please answer all questions with a value between 1 and 7.**  **Part 1: Physician’s Attitudes toward the CDS**  Strongly disagree Strongly agree  1 2 3 4 5 6 7   1. Overall, the CDS has a positive impact on my workload. **6** 2. The CDS is easy to use. **6.5** 3. The CDS reduces diagnostic care errors. **7** 4. I feel that I had adequate training on using the CDS. **7** 5. I believe the CDS improves the quality of my patient care. **7**   **Part 2: Physician’s Satisfaction with the CDS**  Not at all satisfied Very satisfied  1 2 3 4 5 6 7   1. How satisfied are you with the CDS for *receiving relevant alerts when appropriate*? **6** 2. How satisfied are you with the CDS for *receiving relevant information/educational materials*? **7** 3. How satisfied are you with the ease of using the CDS? **6.5** 4. Overall satisfaction with the CDS. **6.5**   Other comments and recommendations for improving the CDS:  _______________________________________________  _______________________________________________ |
| --- |

**Supplemental Figure 3A: CELS and CQL for CDS1**

**Piece of Clinical Logic:**

IF [Venous thromboembolism (VTE)] OR [history of Venous thromboembolism (VTE)] OR [family history of VTE] THEN [Physician should deliver supplemental education materials about VTE prevention. If unavailable, please provide supplemental education materials about VTE prevention in order of preference: talking to physician, information on a piece of paper, talking to nurse, talking to pharmacist, watching a ten minute video on smartphone or television]

| library PopoolaCDS version '1.0.0'  using FHIR version '4.0.1'  include FHIRHelpers version '4.0.1' called FHIRHelpers  include CDSConnectCommonsForFHIRv401 version '2.0.0' called CDS  codesystem "SNOMED": 'http://snomed.info/sct'  valueset "VTE": 'http://snomed.info/sct?fhir_vs=isa/111293003'  valueset "PE": 'http://snomed.info/sct?fhir_vs=isa/59282003'  context Patient  define "AnyVTE":  exists ( [Condition: "VTE"] )  or exists ( [Condition: "PE"] )  or exists ( [ FamilyMemberHistory ] F  where exists F.condition C  where ( C.code in "PE" or C.code in "VTE" ))  define "NoVTE":  not ( "AnyVTE" ) |
| --- |

**Supplemental Figure 3B: CELS and CQL for CDS2**

**Piece of Clinical Logic:**

IF [suspected PE] THEN [Wells criteria]

| **Wells' Score** | |
| --- | --- |
| **Name** | **Value** |
| Clinical signs or symptoms of DVT | 3 |
| PE is the leading diagnosis | 3 |
| Heart rate greater than 100 | 1.5 |
| Recently bedridden for at least 3 days, OR major surgery (requiring general or regional anesthesia) within past 4 weeks | 1.5 |
| Previously diagnosed PE or DVT | 1.5 |
| Hemoptysis | 1 |
| Active cancer (treatment or palliation within past 6 months) | 1 |
| None of the above | 0 |

| library PE_CDS version '1'  using FHIR version '4.0.1'  include FHIRHelpers version '4.0.1' called FHIRHelpers  include CDSConnectCommonsForFHIRv401 version '2.0.0' called CDS  codesystem "LOINC": 'http://loinc.org'  code "WellsLoincCode": '89547-4' from "LOINC"  code "DDimerCode": '48065-7' from "LOINC"  context Patient  define "LastDDimerValue":  CDS.QuantityValue(  CDS.MostRecent(  [Observation: "DDimerCode"] e where  e.effective >= now() - 180 day  )  )  // we cannot use QuantityValue since Well's score units is not standardized  define "LastWellsValue":  CDS.MostRecent(  [Observation: "WellsLoincCode"] e where  e.effective >= now() - 180 day  ).value.value.value  define "NoDDimer":  "LastDDimerValue" is null  define "NoWells":  "LastWellsValue" is null  define "WellLessThan4":  "LastWellsValue" <= 4  define "NotElevatedDDimer":  "LastDDimerValue" < 400 'ng/mL'  define "NoDDimerNoWells":  "NoDDimer" and "NoWells"  define "NoDDimerWellLessThan4":  "NoDDimer" and "WellLessThan4"  define "NotElevatedDDimerNoWells":  "NotElevatedDDimer" and "NoWells"  define "NotElevatedDDimerWellLessThan4":  "NotElevatedDDimer" and "WellLessThan4" |
| --- |

**Supplemental Figure 3C: CELS and CQL for CDS3**

**Piece of Clinical Logic:**

IF [age >=18] AND ([primary incident breast cancer diagnosis of ductal carcinoma in situ (DCIS)] OR [American Joint Committee on Cancer (17) stage I–III invasive cancer]) THEN [surveillance with MRI] OR [surveillance with mammography]

| library WernliCDS version '1.0.0'  using FHIR version '4.0.1'  include FHIRHelpers version '4.0.1' called FHIRHelpers  // These ValueSets rely on reasoning over ICD-10, some vendors have implemented it, for example, OpenEHR:  // https://openehr.atlassian.net/wiki/spaces/spec/pages/378830853/Terminology+in+AQL, see "Example request 5"  // But implicit ValueSets over ICD10 have not been identified as necessary by FHIR yet  // https://terminology.hl7.org/ICD.html#icd-x-implicit-value-sets  // Alternatively, see the attached valuesets.json definition file that is compatible with the CQL Execution Framework  // https://github.com/cqframework/cql-execution  valueset "DCIS": 'http://hl7.org/fhir/sid/icd-10-cm/vs?fhir_vs=isa/D05.1'  valueset "BrCa": 'http://hl7.org/fhir/sid/icd-10-cm/vs?fhir_vs=isa/C50'  valueset "SecondaryCancer": 'http://hl7.org/fhir/sid/icd-10-cm/vs?fhir_vs=isa/C79'  context Patient  define "AnyBrCaExcludingMetastatic":  ( exists ( [Condition: "DCIS"]) or exists ( [Condition: "BrCa"] ) )  and not ( exists ([Condition: "SecondaryCancer"]) ) |
| --- |

**Supplemental Table 1: Analysis of Expert Feedback**

| **Sociotechnical Factors** | **Reviewer Comments** |
| --- | --- |
| Person Factors | Automate all exclusions as much as possible. For instance, use age to exclude minors from applicable recommendations (e.g., age<18). |
| Tool Factors | Link to URLs should be on the header in bright yellow background for increased visibility.  Use definitive verbs such as “Please assess PE risk…”, rather than “Risk should be assessed using…”  Fire combination alerts at the same time, but prioritize them in the design, instead of firing two alerts one after another. |
| Task Factors | Minimize providers’ efforts to review CDS alerts. For instance, use a more efficient way to display the textual data in an initial display, rather than adding a window before a provider can proceed to a next one. |

**Supplemental Table 2: Triggers, Inclusion and Exclusion Criteria, and Logic for CDS Hooks**

| **CDS** | **Triggers** | **Inclusion Criteria** | **Exclusion Criteria** | **Logic to Trigger a CDS Hooks Card** |
| --- | --- | --- | --- | --- |
| CDS1 | Provider orders CTPA | 18 years or older | None | Current or past VTE or PE, Family history of VTE or PE |
| CDS2 | Provider orders CTPA | 18 years or older | Pregnancy, Contrast allergy, Renal dysfunction | D-dimer results and Wells’ criteria (see Supplemental Figure 1B) |
| CDS3 | Provider orders Surveillance Mammogram or Breast MRI | 18 years or older, women | None | Previous history of DCIS or breast cancer* |

CDS: Clinical decision support

CTPA: CT Pulmonary angiogram

VTE: Venous Thromboembolism

DCIS: Ductal Carcinoma in Situ

*Stage 4 breast cancer was not excluded. The CDS Hooks card state that the recommendation only applies to patients with DCIS or Stage 1-3 breast cancer.
